# Supplementary material for: What are the expectations and experiences of a GMH research programme delivered in Bosnia-Herzegovina, Colombia and Uganda? A prospective longitudinal qualitative study
Source: BMJ Open. 2022 Jun 3;12(6):e059590. doi: 10.1136/bmjopen-2021-059590 (PMC9171268; doi:10.1136/bmjopen-2021-059590)
Supplement: Supplementary data [file bmjopen-2021-059590supp001.pdf]

Appendices

Appendix A Topic Guides

Section	Question (Expectations)	Question (Experiences)
Introduction	Please tell me a bit about yourself and your professional role(s)<br>Have you previously worked on international projects, or 'global health' projects?<br>If so, please describe your experience working on such projects?	Please tell me a bit about yourself and your professional role(s)<br>[If appropriate] To what extent did your previous experiences or collaborations help you in this role?<br>[If appropriate] How did this collaboration differ from previous collaborations?
Motivations	What were your motivations for getting involved in the project?	Were these motivations realised during the project?
Understanding the group's aims	From your perspective, what are the key aims of the collaboration?<br>How do you think these aims will be achieved?<br>What things do you feel will be important in achieving these aims?	Now having experienced working on the project, what do you believe to be the most important aim(s)?<br>And do you think any of these aims have been achieved?<br>How did your role help achieve these aim(s)?
General expectations/experiences	What do you expect your participation in the collaboration will involve?<br>Can you share with me any potential advantages of being a part of this collaborarion?<br>Can you share with me any potential disadvantages of being a part of this collaborarion?	Please describe your role within this current collaboration?<br>Can you share with me any advantage you experienced or observed in being a part of this collaborarion?<br>Can you share with me any disadvantage you experienced or observed in being a part of this collaborarion?
Mutual learning	Can you give me an overview of your understanding of the term mutual learning?<br>What do you feel that others may be able to learn from you over the next few years?<br>What do you feel you may learn from others over the next few years?<br>How do you see this process of mutual learning working in practice?	Do you think others learnt from your expertise? What specifically did you learn from others?<br>[If appropriate] Have you been able to use of these skills and experiences in other roles?<br>Do you feel the process of mutual learning took place during the project, and how did this happen in practice?

Closing remarks	Is there anything you would like to add, or anything you feel we have not discussed that may be important?	Is there anything you would like to add, or anything you feel we have not discussed that may be important?

Appendix A Topic Guide

Respondent ID	Country	Position	Expectation interviews	Experience interviews
R-01	Bosnia-Herzegovina	Senior Investigator	✓	✓
R-02	Bosnia-Herzegovina	Researcher	✓	
R-03	Bosnia-Herzegovina	Researcher		✓
R-04	Bosnia-Herzegovina	Researcher		✓
R-05	Colombia	Senior Investigator	✓	✓
R-06	Colombia	Senior Investigator	✓	✓
R-07	Colombia	Coordination/management		✓
R-08	Colombia	Coordination/management		✓
R-09	Colombia	Researcher	✓	✓
R-10	Colombia	Researcher	✓	✓
R-11	Colombia	Researcher	✓	
R-12	Colombia	Researcher	✓	
R-13	Colombia	Researcher		✓
R-14	Colombia	Researcher		✓
R-15	Colombia	Researcher		✓
R-16	Uganda	Senior Investigator	✓	✓
R-17	Uganda	Senior Investigator	✓	✓
R-18	Uganda	Senior Investigator		✓
R-19	Uganda	Senior Investigator		✓
R-20	Uganda	Coordination/management		✓
R-21	Uganda	Coordination/management	✓	✓
R-22	Uganda	Senior Investigator	✓	
R-23	Uganda	Senior Investigator	✓	
R-24	Uganda	Coordination/management	✓	
R-25	Uganda	Researcher		✓
R-26	Uganda	Researcher		✓
R-27	Uganda	Researcher		✓
R-28	Uganda	Researcher		✓
R-29	Uganda	Researcher		✓
R-30	Uganda	Researcher		✓
R-31	Uganda	Researcher		✓
R-32	UK	Senior Investigator	✓	✓
R-33	UK	Senior Investigator	✓	✓
R-34	UK	Senior Investigator	✓	
R-35	UK	Senior Investigator	✓	
R-36	UK	Coordination/management	✓	✓

Rajabzadeh V, *et al. BMJ Open* 2022; 12:e059590. doi: 10.1136/bmjopen-2021-059590

R-37	UK	Coordination/management		✓
R-38	UK	Researcher		✓

Appendix B Participant characteristics

Rajabzadeh V, *et al. BMJ Open* 2022; 12:e059590. doi: 10.1136/bmjopen-2021-059590
