# Supplementary material for: What are the expectations and experiences of a GMH research programme delivered in Bosnia-Herzegovina, Colombia and Uganda? A prospective longitudinal qualitative study
Source: BMJ Open. 2022 Jun 3;12(6):e059590. doi: 10.1136/bmjopen-2021-059590 (PMC9171268; doi:10.1136/bmjopen-2021-059590)
Supplement: Supplementary data [file bmjopen-2021-059590supp002.pdf]

Expectations met		
	**Expectations**	**Experiences**
**Clear, regular transparent communication**	*It's about clear communication, making sure people understand, and that there's no sort of misunderstandings.(R-33 UK Senior Investigator)*	*So even though you're collecting your data, you are always in the know of what other people are doing where it is that they have reached otherwise communication back and forth through emails. And even though the data was being collected locally, it was certainly a collaborative research.(R-17 Ugandan Senior Investigator)*
	You know, you need to be very well informed of exactly what your role is going to be on a project, how much time you're putting in, how much you getting paid for that and what the outcomes are supposed to be, what the indicators are and then you evaluate yourself and make sure that work is done. (R-23 Ugandan Senior Investigator)	*Yes, it has been transparent enough for me. Because when the communication is passed on, sometimes from UK to Uganda, you're copied in from the first communication, you do not necessarily receive second hand like flow through the coordinator (R-30 Ugandan Researcher)*
		Even authorship has been discussed during all these meetings. Then, as the programme went on and we discussed more things, I was delighted to see that it was done fairly. Yeah, it has been transparent. (R-18 Ugandan Senior Investigator)
**Relationships based on mutual respect**	*If a particular group likes communicating in a particular way that you adapt your, your style and it is some sort of negotiation that people have different aims that they want to get out of this. (R-33 UK Senior Investigator)*	*There was, on a personal level, mutual respect acknowledgement for different expertise. (R-32 UK Senior Investigator)*
	But still open towards challenges that and respecting everyone on a similar level the better it will be. Curiosity also helps, not tolerance, but curiosity and respect. (R-32 UK Senior Investigator)	*And then the other one in having a collaboration of course, we made friends we've met people that we didn't know before. We continue to work on things together. So, I guess that was also achieved in terms of creating a collaboration. (R-17 Ugandan Senior Investigator)*
	But ensuring that things work out well, respect for each other, and whatever it is you've agreed to be working on. (R-17 Ugandan Senior Investigator)	

**Language as a barrier**	*Of course, there are many barriers. It begins with the language. In European project meetings, it is also fascinating that after a few hours only the native speakers keep talking […] But the others ones just get tired. And it's difficult to, to negotiate in language that's not your own. (R-32 UK Senior Investigator)*	*And then I guess, disadvantages I think one of the hardest things was communication with each of the teams I guess there's, there was always a language barrier with all the teams. (R-36 UK Coordination/Management)*
**Developing research expertise**	*The research and who are very good doing research and know how to do it and so that's something that's also important for me because many groups do design and try to, to run and research, but they are not very effective. (R-06 Colombian Senior Investigator)*	*I think I've learned a lot of how collaborative research works and what is amazing to me is to see that a in Colombia, we are doing great research (R-15 Colombian Researcher)*
		We had a lot of things to learn how to write protocol or standard operating procedure and things like that, and writing a information sheet […] that was something new for us. (R-03 Bosnian Researcher)
**Publications**	*For my intellectual growth, for my visibility, um, because I'm working in research and academia. When you don't publish then it's like everybody's wondering what you're doing. And so the university has expectations. (R-23 Ugandan Senior Investigator)*	*The first drafts were actually written by the research assistants, but not the drafts didn't have the analysis bit of it. Yes butt it was what we have actually done on the sites.(R-26 Ugandan Researcher)*

Table 1. Additional quotes supporting expectations being met

**Expectations exceeded**		
	**Expectations**	**Experiences**
**Commitment to the research**	*Commitment could be a challenge when collecting the data and documenting this. We have seen it before in some projects where the commitment is not that great (R-16 Ugandan Senior Investigator)*	*I thought that the teams were really eager to to make a difference. I know that the local teams tried their level best to make the research possible. [...] I thought that the teams were really dedicated and so they were big part of the facilitation process of making the research happen. (R-37 UK Coordination/Management)*

	And I think that we must select very good that people here, we have some problems about that. […] So, we must think about that in maybe it, we must select very well the person that they are going to be involved in in these types of projects. (R-05 Colombian Senior Investigator)	*Let's start with the facilitators for conduction of the study. We had a good research team. We had a good administrative team. (R-17 Ugandan Senior Investigator)*
**New research opportunities and extended networks**	The reputation of the academic institution is a necessity proved so it's needs to be done well because this is where we are will representing institution here. (R-34 UK Senior Investigator)	*From my point of view, it is a bit easier, since we're working with already established partners. I think a challenge of GLOBE was sort of from the beginning establishing those partnerships and those working relationships and learning how to work with each partner. Whereas with OLA [new study], we already knew the partners, and we already knew what to expect in terms of how we would work together. (R-36 UK Coordination/management)*
	Whereas a project, like they say we have our network and all the other partners have their networks, I suppose it's about forming those links with those other networks. So we, we set to gain, um, from those experiences and also from those connections. (R-33 UK Senior Investigator)	*Rather than having a completely new study on let's say healers in Colombia, we decided to spread our network. That was a decision. I think it was a good decision. (R-32 UK Senior Investigators)*
	I would like to have a good network, to make more projects in continuing to do being a network not only to have it last three years but maybe to construct a real network between all the universities. (R-05 Colombian Senior Investigator)	

Table 2. Additional quotes supporting expectations being exceeded

**Expectations partially met**		
	**Expectations**	**Experiences**
**Ownership of the research**	*Sometimes partnerships don't do so well because the local people feel like they're not being treated fairly. They don't give you a chance to voice, to be active participants and they are relegated to data collectors (R-16 Ugandan Senior Investigator)*	*So it's not just the UK come and provide training for X, Y, and Z that would help people feel increased a sense of ownership that this is a group that is led by a certain country, but this is a group where we are all in equal partnership and we all have a role to play. (R-33 UK Senior Investigator)*

		We just want you to implement it. I think that it wasn't like that the proposals had to be developed with the input from the UK team, but with a lot of input from the Ugandan team. So I think that also was very good in terms of helping us as learning experience, but also for collaboration. So that there's a sense of ownership on our part as well. Not feeling like it should all be just, you know, them to us. (R-18 Ugandan Senior Investigator)
**Limitations to partnership in designing the interventions**	*So, it's expanding and making the research would be more democratic. So it's not just based on how much money you can get in your own country, but it's increasing collaboration so that more people can be introduced to high quality research and that can bring they own specific contribution to that. (R-33 UK Senior Investigator)*	
	Challenges locally in Uganda and challenges with the partnership. So locally of course the work is going to be done here and uh, that means there needs to be called first of all can clear understanding what it is what that this project is all about. (R-16 Ugandan Senior Investigator)	*And to some extent, they accepted the things I suggested and dismissed the things that were wrong, but I felt that I was listened to, that I was heard. The UK team understood the suggestions I had and if they were good, they were accepted. (R-04 Bosnian Researcher)*
		And when you mentioned everyone, I mean, everyone from the most junior researcher to the PI. It was a beautiful experience coming from a completely different backgrounds that is more hierarchical and more oppressing. This was democratic research platform. (R-01 Bosnian Senior Investigator)
		I feel like we did make the decisions like in terms, if they suggested something and we didn't agree with it, then we would have the final say, even though we didn't necessarily know their context, as well as them and what works. (R-38 UK Researcher)

**Coordination and power dynamics**	*I think my role, it's to keep things moving and making sure we meet deadlines from all funders here, and supporting our partner countries and delivering the studies. (R-36 UK Coordination/management)*	*It's tough because it's like, how equitable can it really be when the money comes from the high-income country? [...] There were points at which the teams would be like happy for the UK team to lead it because they [UK team] were experts in how things are run here and how money was won and how grants were, were achieved and, you know, that sort of thing. (R-37 UK Project Manager)*
	I mean in this project we as the coordinating centre so as a coordinating centre, it's our overall responsibility but I think the more collaboration you can build into that process the better […] if it's just one partner coming in and telling the other partners what to do, that can be quite disempowering (R-33 UK Senior Investigator)	*It was a very, very positive experience because they [UK group] were very supportive all the times, they were available anytime for any need. So they were very professional and they were very supportive in that way. (R-10 Colombian Researcher)*
**Investing in local leadership**	*They are [researchers] going to have an opportunity that many of us did not have to work in international collaborations at the beginning of their careers.[…]I believe that the return will be great too.. (R-06 Colombian Senior Investigator)*	*We do not leave enough money for this career path. These academic roles required to do professional research are simply not there (R-33 UK Senior Investigator)*
**Strengthening research capacity**	*I think the three interventions are subsidiary to that and supposed to be a vehicle whereby relationships and capacity will be developed for the future. (R-35 UK Senior Investigator)*	*I do think that COVID, hasn't helped because it prevented face-to-face contact in the last this last year. And this year, was going to focus on dissemination, grant writing, and ideas generation that's not been able to happen. (R-33 UK Senior Investigator)*
	So the more capacity we have means we're a better research in the area, better quality research, but also would be able to provide very competitive grants that are attracting international funding and be seen as global players, uh, in addressing a challenging issues. (R-16 Ugandan Principal Investigator)	

Table 3. Additional quotes supporting expectations being partially met

Expectations not met		
	**Expectations**	**Experiences**
**Opportunity for innovation**	*But when you look into the health system of the UK and that you have been working a lot on providing psycho-therapeutic interventions, not only the classical intervention. So trying to learn about how to really do that, is inspirational is for me. (R-06 Colombian Senior Investigator)*	*I think that these kinds of studies are a novelty here, it is not very frequent to have these. So this research will bring attention to these interventions. (R-08 Colombian Coordination/management)*
		I mean, we are part of this business that is, I don't think, very innovative […] I hope we had an atmosphere where this was stimulated. (R-32 UK Senior Investigator)

**Mutual learning**	*When we participate in international collaborations we tap into resources and here, I don't mean financial resources […] but rather in the intellectual resources that exist out there (R-16 Ugandan Senior Investigator)*	*So I didn't observe much learning across the groups as much as we very much did try and get them to communicate to each other. I don't feel they did. I felt like there was for the main three partners, like the three separate partners or feeding into us, not feeding into each other. (R-38 UK Researcher)*
	It's about learning. It's about working together. It's about being on the same page. I think all groups have an equal contribution to make. (R-23 Ugandan Senior Investigator)	*Maybe we need some interaction a little bit more in some proposals that come from South to North, not North to South. And , I think that it will be very useful to have at least one meeting every three months, for new ideas of research. Because we have a lot of options, different from the UK. (R-05 Colombian Senior Investigator)*

Table 4. Additional quotes supporting expectations not being met
